# Omics-Based Comparison of Fungal Virulence Genes, Biosynthetic Gene Clusters, and Small Molecules in *Penicillium expansum* and *Penicillium chrysogenum*

**DOI:** 10.3390/jof11010014

**Published:** 2024-12-28

**Authors:** Holly P. Bartholomew, Christopher Gottschalk, Bret Cooper, Michael R. Bukowski, Ronghui Yang, Verneta L. Gaskins, Dianiris Luciano-Rosario, Jorge M. Fonseca, Wayne M. Jurick

**Affiliations:** 1 Food Quality Laboratory, U.S. Department of Agriculture, Agricultural Research Service, Beltsville Agricultural Research Center, Beltsville, MD 20705, USA; 2 Invasive Insect Biocontrol and Behavior Laboratory, U.S. Department of Agriculture, Agricultural Research Service, Beltsville Agricultural Research Center, Beltsville, MD 20705, USA; 3 Innovative Fruit Production, Improvement, and Protection, Appalachian Fruit Research Station, U.S. Department of Agriculture, Agricultural Research Service, Kearneysville, WV 25430, USA; 4 Soybean Genomics and Improvement Laboratory, U.S. Department of Agriculture, Agricultural Research Service, Beltsville Agricultural Research Center, Beltsville, MD 20705, USA; 5 Methods and Application of Food Composition Laboratory, U.S. Department of Agriculture, Agricultural Research Service, Beltsville Human Nutrition Research Center, Beltsville, MD 20705, USA

**Keywords:** apple, blue mold, *Penicillium expansum*, *Penicillium chrysogenum*, metabolomics, secondary metabolites, patulin, transcriptomics, whole genome sequencing, comparative genomics

## Abstract

*Penicillium expansum* is a ubiquitous pathogenic fungus that causes blue mold decay of apple fruit postharvest, and another member of the genus, *Penicillium chrysogenum*, is a well-studied saprophyte valued for antibiotic and small molecule production. While these two fungi have been investigated individually, a recent discovery revealed that *P. chrysogenum* can block *P. expansum-*mediated decay of apple fruit. To shed light on this observation, we conducted a comparative genomic, transcriptomic, and metabolomic study of two *P. chrysogenum* (404 and 413) and two *P. expansum* (Pe21 and R19) isolates. Global transcriptional and metabolomic outputs were disparate between the species, nearly identical for *P. chrysogenum* isolates, and different between *P. expansum* isolates. Further, the two *P. chrysogenum* genomes revealed secondary metabolite gene clusters that varied widely from *P. expansum*. This included the absence of an intact patulin gene cluster in *P. chrysogenum*, which corroborates the metabolomic data regarding its inability to produce patulin. Additionally, a core subset of *P. expansum* virulence gene homologues were identified in *P. chrysogenum* and were similarly transcriptionally regulated in vitro. Molecules with varying biological activities, and phytohormone-like compounds were detected for the first time in *P. expansum* while antibiotics like penicillin G and other biologically active molecules were discovered in *P. chrysogenum* culture supernatants. Our findings provide a solid omics-based foundation of small molecule production in these two fungal species with implications in postharvest context and expand the current knowledge of the *Penicillium*-derived chemical repertoire for broader fundamental and practical applications.

## 1. Introduction

Blue mold decay, caused primarily by *Penicillium expansum*, is a major contributor to pome fruit losses, such as apple or pear, during postharvest storage [1]. Decay is initiated when conidia enter wounds or natural openings in the fruit. Once inside the fruit, the fungi cause watery, brown lesions that radiate from the initial wound. Additionally, mycotoxins like patulin are produced by the fungus during infection. Patulin is a genotoxic, cytotoxic, and carcinogenic compound, thus the mandatory limits in many countries are very low for fruit and fruit products [2,3,4,5,6]. Given that fungal rot negatively impacts the fruit quality and, in cases of mycotoxin producers, renders the fruit unsafe for human consumption, measures to mitigate rot are of utmost importance. However, current industry practices for fungal postharvest decay management are not sufficient in fully preventing blue mold decay. This issue is further compounded by increasing numbers of reports concerning fungicide resistance to all four fungicides utilized in the U.S. apple industry [7,8,9,10,11,12]. Moreover, many countries have lowered the allowable level of fungicide residues on imported fruit thus restricting their use. Additional mitigation strategies are needed to effectively address this growing agricultural challenge.

*P. expansum* causes blue mold decay through numerous virulence factors [1,13]. For example, patulin production impacts virulence, as demonstrated through experiments using genetically defined mutants in *P. expansum* and in pharmacologically-based studies [14,15,16]. Other virulence factors associated with blue mold include polygalacturonases (e.g., PepG1), pH modifiers (e.g., PacC), and effectors (e.g., PePrt, NLP1, Scp) [13,17,18,19,20]. Additionally, many proteins (e.g., Blistering1, LaeA, CreA, SntB, PacC, and VeA) are known to regulate these virulence factors, modulate secondary metabolite production, alter ambient pH, and impact general catabolism [14,20,21,22,23,24,25]. Together, these diverse activities act in an orchestrated manner to facilitate decay in the fruit host.

*Penicillium* spp. occupy different ecological niches and serve as human pathogens, saprophytes, and plant pathogens [26]. Unlike *P. expansum*, *Penicillium chrysogenum* does not cause decay when inoculated into wounded apple fruit [13,27]. *P. chrysogenum* has been exploited by industry for production of the widely used antibiotic, penicillin [28]. Indeed, the penicillin biosynthetic gene cluster was the first fungal biosynthetic gene cluster described, and extensive research has been performed to optimize its production and to understand *P. chrysogenum* metabolism [29].

Development of novel treatments to combat postharvest fungal phytopathogens is critical with the rise of fungicide resistance strains. One increasingly popular approach has been the use of biocontrol organisms and their natural products for the development of new biorationales. Biocontrol agents are successful through a variety of functional mechanisms, including direct microbial antagonism, reduction of nutrient availability and induction of host defense responses [30,31,32]. A recent study by our group found that two *P. chrysogenum* isolates, 404 and 413, were non-pathogenic in apple fruit, and significantly reduced blue mold decay when co-inoculated with two highly virulent *P. expansum* strains, Pe21 and R19 [27]. Because of these findings, there is great appeal to further understand the potential of these two isolates to antagonize *P. expansum*. However, investigation into their small molecule repertoire, toxin production potential, and virulence mechanism is necessary to determine their suitability for use in a postharvest context.

In this study, we investigated two *P. chrysogenum* isolates that cannot cause decay of apple fruit, 404 and 413, at the genomic, transcriptomic, and metabolomic levels in vitro, and compared them with two highly aggressive *P. expansum* R19 and Pe21 strains that cause significant blue mold decay. Through these comparative studies, insights into *P. expansum* virulence, patulin production, and unique genes and metabolites of both species were found. Further, the described strains may be useful for genetic manipulation and exploitation to produce compounds of interest and/or as biocontrol antagonists that can outcompete pathogenic blue mold strains.

## 2. Materials and Methods

### 2.1. Fungal Isolate Propagation and Sample Inoculation

Four fungal strains, *Penicillium expansum* R19, *P. expansum* Pe21, *Penicillium chrysogenum* 404, and *P. chrysogenum* 413 were obtained from previous studies [27,33,34], Each were grown on potato dextrose agar (PDA; Thermo Fisher Scientific, Waltham, MA, USA) plates for 7 days at 25 °C. Conidia were washed from the plate using Tween-treated water (0.1% Tween-20, Sigma Aldrich, St. Louis, MO, USA) and adjusted to 1 × 10^6^ conidia/mL. Flasks containing 50 mL of potato dextrose broth (PDB; Thermo Fisher Scientific, Waltham, MA, USA) were individually inoculated with 100 µL of conidial suspension. Cultures were grown for 7 days, shaking at 25 °C (125 RPM; Innova 42R, Eppendorf, Hamburg, Germany). Each flask was vacuum filtered through a sterile 0.2 µM cellulose acetate filter. The mycelial mat was aseptically transferred to a 50 mL conical tube. Filtered broth was transferred separately to 50 mL conical tubes. Mycelial mats were weighed for each sample, and pH was measured for each broth culture (Figure S1). All broth and mycelial mats were immediately submerged in liquid N_2_ and stored at −80 °C. Broth samples were subsequently used for metabolite extraction for LC-MS and HPLC, whereas the mycelial mats were used for both DNA and RNA extractions for genomic DNA and RNA sequencing.

### 2.2. Fungal Genomic DNA Extraction

High-quality genomes of Pe21, 404, and 413, were produced to compare to each other and to the existing *P. expansum* R19 genome (NCBI GCA_004302965.1). Mycelial mats of Pe21, 404 and 413 were ground to a fine powder using a sterile mortar and pestle with liquid N_2_. DNA was extracted using a phenol-chloroform extraction method (Pacific Biosciences, Menlo Park, CA) with an added RNAse (Thermo Fisher Scientific, Waltham, MA, USA) treatment for 30 min at 37 °C. DNA was resuspended in EB buffer, then visualized for quality via agarose gel electrophoresis (0.8% agarose, 90 volts for 45 min). Samples were quantified using Qubit (Invitrogen Qubit dsDNA H5, Waltham, MA, USA) And stored at −80 °C.

### 2.3. Oxford Nanopore Technology Sequencing

High-molecular weight (HMW) DNA obtained from the isolate samples of Pe21, 404, and 413 was used to prepare Oxford Nanopore Technologies (ONT; Oxford, UK) sequencing libraries using the V14 kit enabled with duplex reads (SQK-LSK114). Generated libraries were evaluated for quantity and quality using a TapeStation 4150 (Agilent, Santa Clara, CA, USA) with the genomic DNA ScreenTape and ladder for fragments >60 Kb. Libraries with >10–20 fmols of 50 Kb or greater fragments were chosen for sequencing on a ONT MinION device. All sequencing and preparation was done in-house. Each respective sample was sequenced on its own ONT R10.4.1 flow cell for ~72 h with reads <1000 bp filtered from the output using ONT sequencing software MinKNOW (v23.04.6). Real-time basecalling was disabled and POD5 files were the chosen output format. The resulting POD5 files were used as input into ONT pytorch basecaller, Dorado (v0.3.3), using the high accuracy (HAC) basecalling model dna_r10.4.1_e8.2_400bps_hac@v4.2.0 with default settings for the Pe21 sample and the super accurate (SUP) basecalling model dna_r10.4.1_e8.2_400bps_sup@v4.2.0 with default settings for the 404 and 413 samples. Additionally, all POD5s were rebasecalled for duplex reads using the Dorado duplex function. The resulting output bam files were sorted for duplex and simplex reads using samtools (v1.7) and converted into fastq files [35]. The fastq files were then evaluated for read length and base pair quality calls using NanoPlot (v1.40.0) [36] and then processed for adapter sequences using Porechop (v0.2.4; https://github.com/rrwick/Porechop (accessed on 1 October 2023)) with default settings. Lastly, the adapter-removed fastq files were additionally filtered for reads ≥20 kb using NanoFilt (v2.8.0) [37].

### 2.4. Fungal Total RNA Extraction

Mycelial mats from four vacuum filtered liquid cultures of R19, Pe21, 404, and 413 were flash-frozen in liquid N_2_, then ground with mortar and pestle. Each sample (approximately 0.1 mg/ powdered sample) was then processed with the TRIzol reagent protocol (Invitrogen) with slight modification. Briefly, a 25:24:1 phenol:chloroform:isoamyl alcohol (PCI; Sigma-Aldrich, St. Louis, MO, USA) step was added after the initial separation, where the aqueous layer from the TRIzol separation step was mixed 1:1 with PCI, incubated 5 min at 4 °C, centrifuged at 13,500× *g* for 15 min, and aqueous phase transferred for subsequent precipitation and washing steps per manufacturer’s protocol. RNA was dissolved in 50 µL DEPC water and heated for 30 min at 65 °C. Initial quantification and quality of each sample was performed using a Nanodrop spectrophotometer (Thermo Fisher Scientific, Waltham, MA, USA) and by agarose gel electrophoresis (1% agarose, 90 volts, 30 min). Each sample was then stored at −80 °C until sequenced.

### 2.5. RNA Sequencing

RNA samples were sent for RNA sequencing at Novogene (Sacramento, CA, USA). Sample quality was assessed with Agilent 5400, then library prep (polyA enrichment), and finally short read (150 bp reads) paired-end sequencing was performed with a NovaSeq platform. Each strain had four individual RNA sequencing (RNA-seq) libraries generated.

### 2.6. Fungal Genome Assembly and Annotation

The sequencing provided opportunity to evaluate multiple assembly methods using the emergent duplex reads, simplex reads, and combined read types. For this, the focus was on 404 as it was the first sample sequenced. The first assembly was completed with Flye (v2.9.2) [38] using the --nano-hq parameter and only the simplex reads used. We also ran Flye in a module-based approach with the initial assembly from simplex reads used as input followed by the Flye -resume-from-polish module with the duplex reads. The second assembly was with HiFiasm (v0.19.5) [39] using the duplex reads the HiFi input and the simplex reads in the -UL input for ultra-long reads. The third method utilized Verkko (v1.4.1) [40] with the duplex reads used as --hifi inputs and the simplex reads as --nano inputs. If the assembler required estimated genome size, a value of 32.4 Mb was used as input. Comparisons of the resulting assemblies were performed using genometools seqstat (v.1.6.2; http://genometools.org/ (accessed on 1 October 2023)).

For the 413 genome, we followed the same approach of using Flye (v2.9.2), followed by Flye -resume-from-polishing step. However, the assembly was more fragmented than 404 and thus we performed homology-based scaffolding. The homology-based scaffolded utilized the RagTag software (v2.1.0) [41] with the 404 assembly used as a guide. For the Pe21 genome, ONT sequencing yielded few duplex reads and thus was assembled only from the simplex reads using Flye. We also used the homology based scaffolding approach for Pe21 with a separate assembly of a mutant Pe21 strain Pe_T1 (unpublished) followed by RagTag patch to gap fill using the NCBI GCA_000769735.1 accession (Pe21; synonym D1 strain) [34]. Genome and scaffolding stats were assessed using genometools seqstat and BUSCO (v5.3.1) [42] with the eurotiales_odb10 database. We further filtered all assemblies for sequencing artifacts and contig fragments with lengths <100 Kb and coverage that was >2 fold of the four longest contigs using seqtk (v1.4; https://github.com/lh3/seqtk (accessed on 1 October 2023)) prior to scaffolding.

In addition to the three genomes of Pe21, 404, and 413, we additionally set out to annotate the existing *P. expansum* R19 genome (NCBI GCA_004302965.1). The aim was to generate annotations for all four assemblies using the same extensive approach previously used for *Fusarium* and *P. fuscoglaucum* genomes [43,44]. The resulting assemblies were first annotated for transposable elements and other repetitive features using RepeatModeler (v.2.0.1) [45] and RepeatMasker (v.4.1.1) [46] hosted on the GenSAS server (v6.0) [47]. GenSAS’ consensus repeat annotation was then invoked to generate a GFF file that combined the features identified by both RepeatModeler and RepeatMasker along with soft masked and hard masked fasta files. The RNA-seq files were first processed to trim adapter sequences and low-quality bases using fastp (v0.23.2) [48]. The processed reads files were then mapped to the hard masked sequences for each respective assembly using HiSAT2 (v2.2.1) [49] using the –max-intronlen setting of 2000 bp. The resulting alignment sam files were then sorted, indexed, and merged using SAMtools. The merged mappings were then used as input in StringTie2 de novo transcriptome assembled using default settings (v2.2.0) [50]. The resulting annotation file (gtf) was used as input into gffread (v0.12.7) [51] along with the hard masked sequence file to extract transcript sequences to serve as pseudo expression sequence tags (ESTs) evidence during ab initio gene annotation. For the ab initio gene annotation, we used the Maker (v.3.01.03) [52] pipeline with the pseudo-EST and protein evidence from *P. camemberti* from NCBI (GCA_000513335.1) [53] as inputs in the first round of annotation for Pe21 and 404. Following the first round of evidence-based Maker annotation, we trained Augustus (v.3.4.0) [54] and SNAP (v2006-07-28) [55]. We then ran a second round of Maker using the trained Augustus and SNAP models for ab initio annotation followed by a second round of training for each gene predictor. A final round of Maker annotation was performed, and the resulting predicted annotations were assigned functional annotation using BLASTP (v2.14.0) [56] and Interproscan (v5.61-90.3) [57]. For R19 and 413, we employed only a single round of Maker annotation using the Augustus and SNAP models generated for Pe21 and 404, respectively. We also included strain-specific pseudo-ESTs and the same *P. camemberti* protein fasta as additional evidence into the Maker pipeline. Non-coding genes (e.g., rRNA, tRNA) were annotated using the Infernal software suite (v1.1.4) [58] with default inputs. Lastly, annotation quality and descriptive statistics were calculated using BUSCO with the eurotiales_odb10 database and AGAT (v0.9.1; https://github.com/NBISweden/AGAT#publication-using-agat (accessed on 1 October 2023)), respectively.

### 2.7. Comparative Genomics

To determine regions of synteny and collinearity between the four assembled genomes, the python MCScanX pipeline (https://github.com/tanghaibao/jcvi/wiki/MCscan-(Python-version) (accessed on 1 October 2023)) [59] approach was employed. Here, the annotation gff files for each genome was converted to bed format using the MCScanX python version jcvi.formats.gff function. We then ensured that the fasta transcript files from each assembly were also of a compatible format using the jcvi.formats.fasta function. Then, synteny was classified in a pairwise fashion using the jcvi.compara.catalog function for R19 vs. Pe21, Pe21 vs. 404, and 404 vs. 413, then performed the jcvi.compara.synteny function using the --minspan = 30 and --simple parameters. Matching layout and seqids files were then created as directed in the protocol (https://github.com/tanghaibao/jcvi/wiki/MCscan-(Python-version)#workflow (accessed on 1 October 2023)). Finally, figures were generated using the jcvi.graphics.karyotype function. Additionally, Orthofinder (v2.5.5) was used to identify orthogroups between the four assemblies [60]. Orthogroups were identified by using the annotated protein fasta files generated during genome annotation as input into Orthofinder (v2.5.5) while invoking the -M msa command. Annotated virulence genes from *P. expansum* were compiled from the primary literature (Table S1) and fastas were obtained from their respective NCBI accessions. These fastas were concatenated in a single fasta and used to identify their corresponding transcripts in the R19 transcript annotation using blastn. The blastn results were then used to identify single copy orthologues for each virulence gene across the four *Penicillium* samples using the Orthofinder results.

For secondary metabolite cluster identification, antiSMASH (v7.0.1) was run for each of the new 404, 413, and Pe21 genomic fasta files, and the re-annotated R19 genome (see Genome Annotation) [61]. Every “extra feature” was selected and executed using the ‘relaxed’ parameters within the fungal database.

### 2.8. Gene Expression Analysis

To compare gene expression profiles for each of the four samples, we used the RNA-seq mappings generated during genome annotation as input into StringTie2. StringTie2 was executed with the -B and -e commands to calculate expression statistics. The reference annotation for each respective sample was used as the -G input into the StringTie2 command. The resulting gtf files were then used as input in the PrepDE.py3 script provided by StringTie2 to extract hypothetical read counts per transcript information. Transcripts per million (TPM) measurements were then extracted as text files and used as input into R (v4.3.2) [62] for transcriptome visualization. TPM for each ortholog was then visualized using the heatmap function in R.

### 2.9. Metabolite Extraction

Filter-sterilized (0.2 µm) spent growth medium (50 mL; see ‘Fungal isolate propagation and sample inoculation’) from three independent cultures of Pe21, R19, 404, and 413 and uninoculated medium (five aliquots) was processed as previously described [21]. Each were centrifuged for 30 min at 15,000× *g* (rotor JA-12, Beckman Coulter, Brea, CA, USA). Chloroform was added to 3 mL medium at a 1:1 ratio before centrifuging again at 10,000× *g* for 10 min. The supernatant was transferred to a tube containing 3 mL 60% methanol:ddH_2_O and vortexed for 2 min (Vortex Genie 2, Scientific Instruments, Schaumburg, IL, USA), then mixed with 5 mL of ethyl acetate. The top organic layer was transferred to a 15 mL conical tube and evaporated to dryness with continual flow of nitrogen gas using an N-EVAP nitrogen evaporator (Organomation, Berlin, MA, USA), then stored at −80 °C until resuspension.

### 2.10. Metabolomic Analysis

Three 404, 413, Pe21, and R19 sample residuals from the metabolite extraction were resuspended in 100 µL 50% methanol/0.1% formic acid. Twenty µL from each sample were pooled to make a quality control (QC) sample. Three samples of PDB without fungus were processed similarly but were not included in the QC. Five µL of the samples were separated on a 40 °C heated, 150 × 2.1 mm Hypersil GOLD VANQUISH HPLC column with 1.9 µm particles (Thermo Fisher Scientific, Waltham, MA, USA) coupled to a Vanquish HPLC pump (Thermo Fisher Scientific, Waltham, MA, USA) controlling a 10-min linear gradient from 0% to 95% acetonitrile and 0.1% formic acid at a flow rate of 0.2 mL per minute. Eluent was electrosprayed at 3.5 kV positive polarity into an Exploris 240 mass spectrometer (Thermo Fisher Scientific, Waltham, MA, USA) using an internal mass calibrant. Sheath gas was 35, auxiliary gas was 7, and sweep gas was 1 (arbitrary units). The ion transfer tube temperature was 325 °C and the vaporizer temperature was 275 °C. Advanced peak determination, mild trapping, and internal mass calibration was enabled. Default charge state was one and the expected peak width was 6 s. AcquireX software (accessed 1 July 2023) was used to create a background ion exclusion list from a blank sample consisting of 50% methanol/0.1% formic acid and an inclusion ion list from the QC sample [63]. Subsequently, five injections of QC were performed to generate MS^2^ spectra. After each of those injections, the resolved ions were appended to the exclusion list for the subsequent injection. Survey scans were recorded in the Orbitrap at 60,000 resolution over a mass range of *m*/*z* 70–800. The RF lens was 70%. Monoisotopic precursor selection was enabled, the minimum intensity was 5000, charge states were filtered to one and automated dynamic exclusion was enabled. Twenty precursors selected within a 1.0 Da isolation window were fragmented by high energy collision-induced dissociation (30%, 50%, 70% normalized stepped collision energy), and fragment ions were resolved in the Orbitrap at 30,000 resolution. Next, all test samples were analyzed alongside four intermittent injections of QC and blank consisting of 50% methanol/0.1% formic acid. Survey scans were recorded in the Orbitrap at 120,000 resolution over a mass range of *m*/*z* 70–800. The RF lens was 70%. The samples were also analyzed in negative ion mode at −2.5 kV but with the same other settings. These mass spectrometry data files can be retrieved from massive.ucsd.edu (MSV000095703). All samples were prepared and run in-house.

Result raw files were analyzed with Compound Discoverer version 3.3 (Thermo Fisher Scientific, Waltham, MA, USA). The ChromAlign node was used to align chromatographic peaks in all files to a QC replicate. The Detect Compounds node was used with 2 ppm mass tolerance, 10,000 minimum peak intensity, at least five scans per peak, 0.25 min maximum peak width, and compound detection of [M + H]^+1^ ions (positive ion mode) or [M − H]^−1^ ions (negative ion mode) to create peak areas. The Group Compounds node was used with 2 ppm mass tolerance, 0.25 min retention time (RT) tolerance, and a peak rating threshold of four for a minimum of five files. The Fill Gaps node was used with 2 ppm mass tolerance, the SERRF QC Correction node was used with 75% QC coverage to normalize the peak area results, and the Mark Background Components node was enabled. The Search mzCloud node was used to compare MS2 spectra to all compound classes at a precursor mass tolerance of 5 ppm and fragment mass tolerance of 5 ppm. The Search mzVault node was used to compare MS^2^ spectra to the NIST_2020_MSMS High Resolution library at fragment mass tolerances of 10 ppm. The Predict Compositions nodes were used with 2 ppm mass tolerances. The software performed a one-way ANOVA to estimate compound peak area statistical differences and used the Benjamini and Hochberg method to estimate the false discovery rate (FDR). Filtering of results was performed to limit background ions, to use normalized peak areas, and to require MS^2^ supporting spectra. Results were further filtered to require one match score greater than or equal to 70, R19/PDB compound peak area ratios greater than or equal to 2 log_2_ (with a similar increase in Pe21/PDB and almost no increase or a slight increase/decrease for both 404/PDB and 413/PDB) and have a FDR less than 5%. This yielded compounds with substantial increases for R19/404. A second result list was generated with 404/PDB compound peak area ratios greater than or equal to 2 log_2_ (with a similar increase in 413/PDB and almost no increase or a slight increase/decrease for both R19/PDB and Pe21/PDB). This yielded compounds with substantial increases in 404/R19. Results were examined by hand and discarded if the chromatographic peaks were substandard or if the MS^2^ identifying spectrum did not fall within the assigned chromatographic peak area.

### 2.11. HPLC-High Resolution Mass Spectrometry

Filtered samples of supernatant were analyzed for patulin using an Agilent 1290 UHPLC coupled to an Agilent 6545XT hybrid quadrupole/time-of-flight mass spectrometer (Santa Clara, CA, USA). Samples were prepared and run in-house. Separations were performed on a Peek-lined AdvanceBio MS Spent Media column (2.1 × 100 mm, 2.7 µm) with a binary solvent gradient having an overall flow of 250 µL/min. Solvent A was 10 mM ammonium acetate in HPLC-MS grade water (Sigma Aldrich, St. Louis, MO, USA) and solvent B was 95:5 methanol: 200 mM ammonium acetate. Samples were separated using the following gradient: 0–5 min, 2% solvent B; 3–3.5 min, 10% solvent B; 3.5–4 min, 100% solvent B; 4–8 min, 100% solvent B; 8–8.2 min, 2% solvent B. Data were collected over a mass range of 100–1000 Da. Patulin was identified using three ions previously described [64]: *m*/*z* = 155.0339, [M + H]^+1^; *m*/*z* = 169.0495, [M—H_2_O + CH_3_OH]^+1^; *m*/*z* = 137.0233, [M—OH]^+1^. Source parameters were optimized to increase detection of *m*/*z* = 137.0233, which was used as the quantitating ion, with *m*/*z* = 169.0495 used as a qualifying ion (Table S2). Quantitation was performed using a matrix-matched external calibration set with patulin concentrations of 0.5, 1, 5, 10, 25, 100, and 250 µg/mL. The calibration data was collected in triplicate and fit with second-order polynomial.

## 3. Results

### 3.1. Fungal Genome Sequencing and Assembly

Whole genome sequencing using nanopore technology was used to obtain improved quality of the available genomic resources for *Penicillium chrysogenum* 404, 413, and *Penicillium expansum* Pe21. We averaged 9.9 Gb of sequence for each sample with a mean phred score of Q16.9 (Table S3). Following filtering, the resulting sequence datasets had an average size of 2.2 Gb with read N_50_ between 22 Kb and 32 Kb. We also performed duplex basecalling where each dataset yielded between 0.34 Gb to 1.1 Gb of sequence. The duplex reads had an average N_50_ of 8175 bp and mean phred score of Q24.8. The duplex read output was 5% to 10% of the total sequence data (e.g., duplex efficiency) (Table S3). The duplex datasets were deemed too low in sequence amount for accurate de novo assembly. For the filtered simplex data, we achieved a minimum of 48× coverage for a genome of an estimated 32.4 Mb in length (Table S3). This resulting sequence was of sufficient coverage to proceed with de novo genome assembly. The 404-sequencing yielded sufficient coverage of duplex only reads to attempt duplex-based assembly and thus was a proof case for evaluation of different assembly methods and inputs. We utilized Flye with simplex only reads for the initial assembly and employed the Flye polishing module with the duplex reads. The initial N_50_ read length was 5.4, 9.1, and 6.4 Mb for Pe21, 404, and 413 respectively (Table 1).

Comparing these two resulting assemblies, the polishing resulted in some “scaffolding” of shorter contigs and slight decreases in total length and N_50_ (Table 1). This suggests that the polishing with duplex reads was able to correct and place some contigs to increase the contiguity of the assembly. We also performed mixed assemblies with HiFiasm and Verkko using the duplex reads as a pseudo HiFi input. In both cases, the resulting assemblies were more fragmented suggesting Flye assembled superior genomes for this study (Table 2). We decided to move forward with Flye and duplex polishing as our primary assembly method except for Pe21 where not enough duplex reads were generated. We additionally employed some scaffolding and gap filling (i.e., patching) for the 413 and Pe21 assemblies.

Our final assemblies, following the polishing, scaffolding, and/or patching, resulted in highly contiguous genomes (Table 1). The assembly for Pe21 had a total length of 32.4 Mb, which is similar to the previously published R19 strain for *P. expansum* [65]. Moreover, GC content between the two only differed by 0.2%. Furthermore, BUSCO scores for both *P. expansum* genomes were above 96.0% complete with the Pe21 being 0.5% higher than R19 along with lower fragmented and missing BUSCOs. For the two *P. chrysogenum* genomes, 404 was more contiguous after the initial assembly step and thus served as a reference for the scaffolding of 413. Both assemblies were 32.5 Mb in length but 413 increased by another 100 Kb following scaffolding. The N50 for both post processed assemblies were nearly identical with values of 9.11 Mb and identical BUSCO scores. Overall, the assemblies for each species and two strains were highly similar in quality and contiguity, suggesting our assembly approaches were sound and effective (Table 1).

### 3.2. Penicillium spp. Genome Annotation

Using the three high quality genomes we assembled, in addition to the unannotated *P. expansum* R19 genomic resource available (NCBI accession PRJNA497398), we set out to thoroughly annotate each genome independently. For the *P. expansum* strains, we identified 9740 and 10,536 features of repetitive nature (e.g., transposable elements, simple repeats) (Table 3). Similarly, the *P. chrysogenum* yielded 10,111 to 10,400 repetitive elements. However, the total length of the repeats was >1 Mb more in *P. chrysogenum* than *P. expansum.* For the gene annotation, *P. expansum* had between 11,326 and 11,911 protein coding genes, whereas *P. chrysogenum* had between 12,246 and 12,774 protein coding genes. Average gene lengths between all the four assemblies were similar, with a range of 1661 bp for Pe21 to 1890 bp for R19. The average number of exons per gene was also similar with 3.2 for *P. expansum* and 3.1 for *P. chrysogenum.* Overall, all four genomes were thoroughly annotated with a BUSCO completeness score above 98.1% and a max of 1.2% missing BUSCOs (Table 3).

### 3.3. Macro- and Micro-Synteny in Penicillium Species

**Figure 1:**
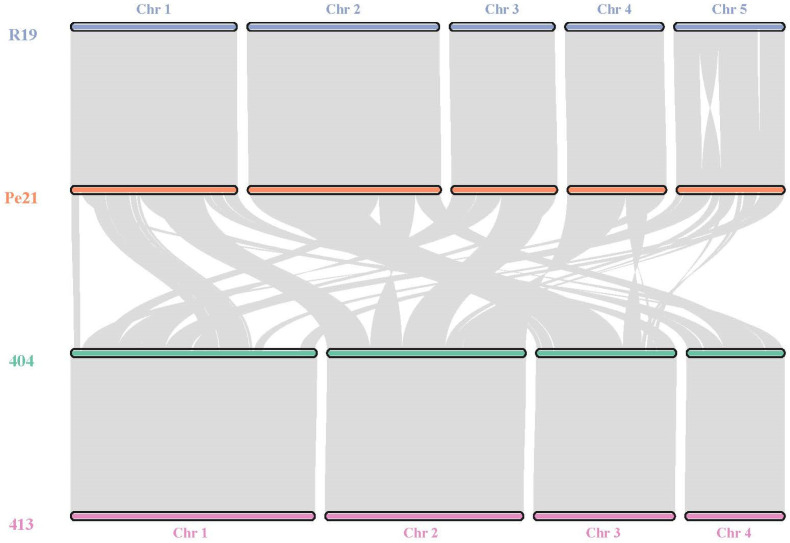
Genome macrosynteny between *P. expansum* R19 (blue), *P. expansum* Pe21 (orange) *P. chrysogenum* 404 (green), and *P. chrysogenum* 413 (pink) chromosomes (chr). Gray lines represent regions of synteny and connect to similar areas across chromosomal regions.

To evaluate the synteny and collinearity of the genomes both inter- and intra-specifically, we performed ortholog block identification using MCScanX. The resulting analysis demonstrated high intra-specific collinearity between the strains, whereas the interspecific synteny was highly decayed between the two species (Figure 1). The *P. chrysogenum* genomes were each composed of four chromosomes, whereas the *P. expansum* genomes spanned five, matching previously published numbers and sizes for each species [66,67]. Further, it appears the two *P. expansum* isolates also have an inversion in the fifth assembled contig. Many blocks of colinear genes were maintained between the species but were inverted, translocated, and/or rearranged to the point where chromosomes were no longer homologous (Figure 1).

### 3.4. Secondary Metabolic Gene Clusters

The program antiSMASH was used to discover secondary metabolic (SM) gene clusters that are present in the four genomes. In strains 404 and 413, 56 SM clusters were identified, of which 21 were associated with known biochemical pathways (Table 4).

The Pe21 and R19 genomes had a total of 66 and 67 secondary metabolite clusters, respectively, with 28 and 27 annotated with known metabolite production. The identified biosynthetic gene clusters were classified as (or like) non-ribosomal protein synthetases (NRPS), type 1 polyketide synthases (T1PKS), indoles, terpenes, or fungal ribosomally synthesized and post-translationally modified peptides (fungal-RiPP-like). Many instances of identified clusters showed only partial matches, suggesting the potential for metabolite production may be varied despite some nucleotide sequence homology. Pe21, R19, 404, and 413 only matched 7, 6, 8, and 8 clusters at 100%, with lowered stringency of 50% cluster matches for 18, 17, 14, and 14, respectively. Of the annotated clusters, seven were present in all four isolates above 50%. Only three SM clusters, andrastin A, choline, and YWA1 (2,5,6,8-tetrahydroxy-2-methyl-2,3-dihydro-4*H*-naphtho [2,3-*b*]pyran-4-one), had a 100% match in all four isolates, demonstrating that most of the secondary metabolic capacity is distinct between the two species. For example, the *P. expansum* isolates had clusters with at least 50% identity for AbT1, communesins, citrinin, clapurines, gliovirin, phomasetin, Hex-pks1 polyketide, and neosartorin. The *P. chrysogenum*-specific SM clusters included conidiogenone, chrysogine, macrophorin A, PR-toxin, sorbicillin, penicillin, and metachelin/dimerumic acids. Two isolate-specific SM clusters were also exclusively detected in the *P. expansum* genomes, which were ACT-Toxin II in Pe21, and cyclo-peptides in R19.

### 3.5. Conserved Expression for Virulence Gene Orthologues in Penicillium spp.

**Figure 2:**
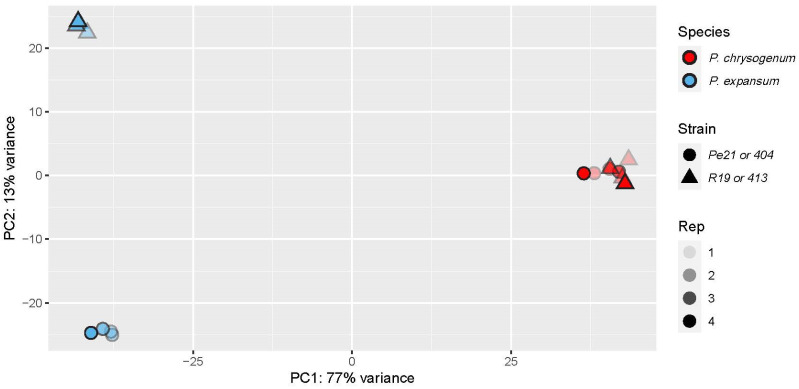
Principal Component analysis of transcriptomic data for each *Penicillium* spp. isolate. *P. chrysogenum* isolates 404 (red circles) and 413 (red triangles), *P. expansum* isolates R19 (blue triangles) and Pe21 (blue circles) across four replicates.

In addition to transcriptomic guidance to aid high-quality genomic annotation, we sought to compare transcriptomic profiles across the species to further understand their similarities and unique traits. A full list of conserved, single-copy gene orthologues was generated across the four *Penicillium* genomes. The expression of the orthologues across all isolates showed species-specific clustering with a principal component analysis (PCA; Figure 2). Further, 404 and 413 isolates clustered much closer together than the two *P. expansum* isolates, demonstrating less gene expression variability within the *P. chrysogenum* isolates in culture.

### 3.6. P. chrysogenum Isolates Share P. expansum-Associated Virulence Gene Orthologues

A select number of *P. expansum* genes have been experimentally verified via gene functional analysis to contribute to virulence during pome fruit decay (Table S1). Therefore, the 404, 413, R19, and Pe21 genomes were comprehensively searched for virulence gene orthologues to investigate the observed non-virulent phenotype of *P. chrysogenum* in apple fruit. Thirty-nine single-copy orthologues were found within each of the four genomes (Table S1). The *P. chrysogenum* genomes did not have any orthologues for four genes encoding PeLysM5, PeLysM8, PeLysM11, and PeLysM16. PeLysM7 was found in each genome except in 404. The remaining 12 orthologues were identified with multiple copy numbers within at least one of the genomes. Expression (log2 TPM) of the 39 single copy orthologues was evaluated (Figure S2). Most of the genes were similarly expressed in culture within and between species, with little transcript detected for the gene encoding PeLysM15 in all isolates, and PeLysM14 in *P. expansum* isolates grown in culture (Figure S2).

### 3.7. Penicillium spp. Produce an Array of Metabolites In Vitro

**Figure 3:**
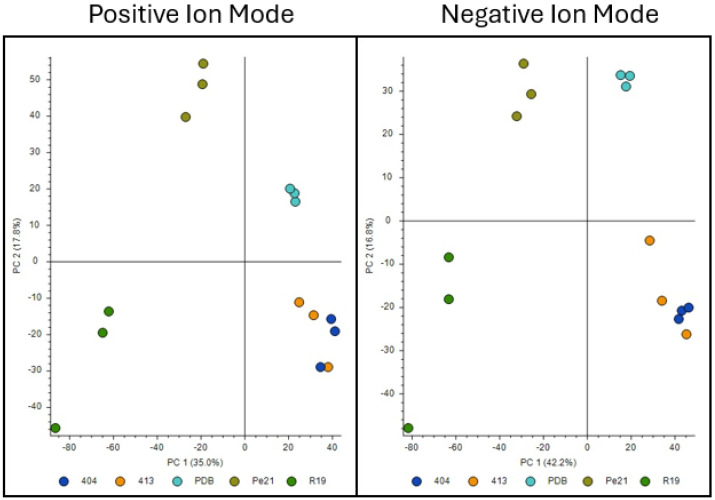
Principal Component analysis of metabolomic data for each *Penicillium* spp. isolate. *P. chrysogenum* isolates 404 (navy blue) and 413 (orange), *P. expansum* isolates R19 (green) and Pe21 (brown), and the PDB control samples (teal). Samples were analyzed in both positive ion mode (**left**) and negative ion mode (**right**).

Untargeted metabolomic profiling was performed on the spent liquid culture broth for each isolate. Across all samples, 4478 and 4448 compound features (by *m*/*z* and RT) were detected in positive and negative ion modes, respectively. PCA revealed species-level differences in the metabolomic profiles like the transcriptomic PCA, with 404 and 413 clustering tightly with each other but separately from R19 and Pe21 (Figure 3). These patterns indicate congruence between gene expression and small molecule production at a global level.

Individual compounds were selected based on their spectral library match scores (>70) and accumulation differences (peak area significance *p* < 0.05) compared to the PDB control samples and higher abundance in one or both *P. chrysogenum* (Table 5) or *P. expansum* isolates (Table 6).

Many of the compounds identified can be grouped into one or more category based on potential biological activity. For example, multiple abundant compounds in *P. chrysogenum* have possible pharmacological activity, including anticancer and anti-inflammatory compounds like luteolin, rubiadin, citreorosein, and N-acetyltyramine, or other potential functions, like 6-methoxy-2-oxo-2H-1-benzopyran-3-carboxylic acid, a diuretic. Another subset in *P. chrysogenum* was previously shown to be antibacterial, antiviral, or antifungal, such as penicillin G, 5-methyl-L-uridine, 5-methylcytidine, and scopoletin. It should be noted that many of the preceding compounds have several known biological activities that may depend on biological context.

In *P. expansum*, a completely different set of antimicrobials was produced, including the mycotoxin patulin, 3,5-dimethylorsellinic acid, perillic acid, 4-methoxycinnamic acid, and gentisic acid. A subset of compounds produced by *P. expansum* may also act as plant metabolites, including gallic acid, gentisic acid, and an isomer of indole-3-acetic acid (IAA).

### 3.8. Linking Patulin and Penicillin G Gene Clusters, Expression and Metabolite Production

**Figure 4:**
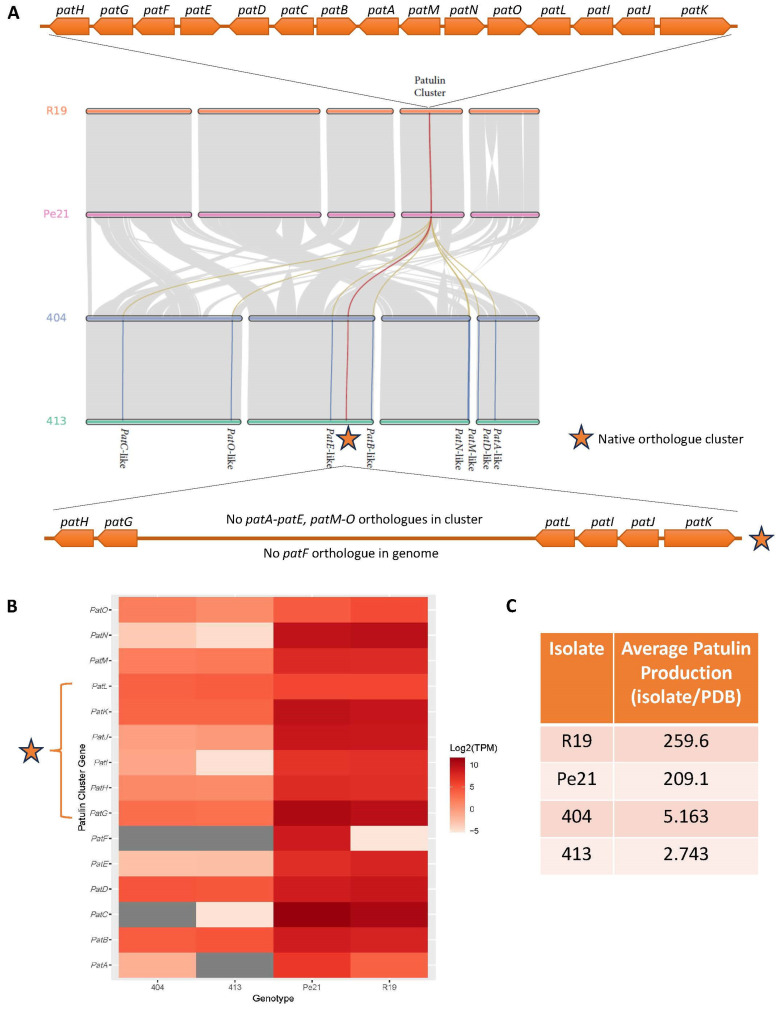
Patulin genomic, transcriptomic, and metabolomic presence in each *Penicillium* isolate. (**A**) Complete 15-gene patulin biosynthetic gene cluster in *P. expansum* found localized to one location in R19 and Pe21 (top, orange line). In 404 and 413, the native orthologous cluster only contains 6 genes (bottom, star), with the remaining orthologues found across the four chromosomes (blue lines). (**B**) Expression (Log2 TPM) of each patulin cluster gene orthologue across the four isolates. Darker red indicates higher expression, and gray means transcript levels were below the threshold. TPM = transcripts per million. (**C**) Average patulin production detected in each isolate over the average potato dextrose broth (PDB) control. Values are average peak area ratios across replicates.

**Figure 5:**
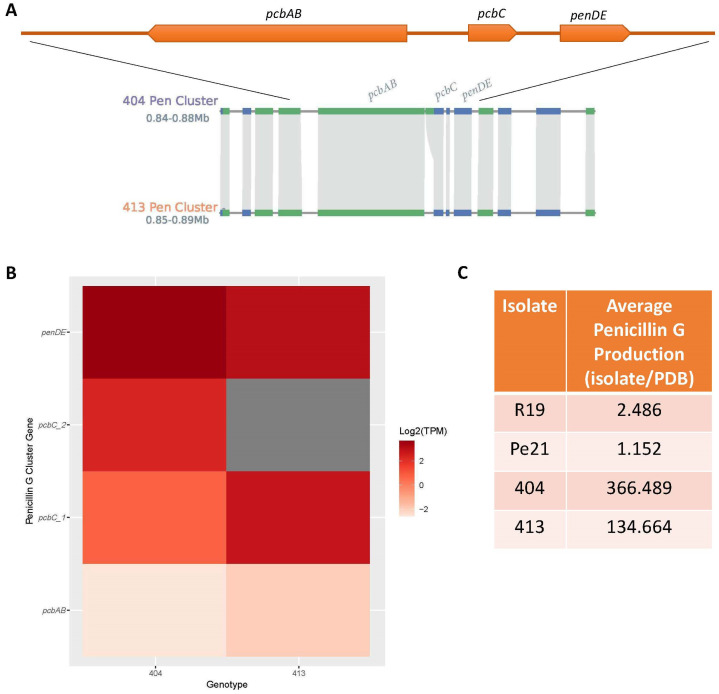
Penicillin G genomic, transcriptomic, and metabolomic presence in each *Penicillium* isolate. (**A**) Penicillin G biosynthetic gene cluster in *P. chrysogenum* (top, orange line) chromosome 1, which were absent in R19 and Pe21. The 404 *pcbC* gene shows a larger segment that suggests a tandem repeat compared to the 413 genome. (**B**) Expression (Log2 TPM) of each penicillin G cluster gene orthologue across the two isolates. Darker red indicates higher expression, and gray means transcript levels were below the threshold. TPM = transcripts per million. (**C**) Average penicillin G production detected in each isolate over the average potato dextrose broth (PDB) control. Values are average peak area ratios across replicates.

The biosynthetic pathways for penicillin G, produced by the *P. chrysogenum* isolates, and patulin, produced by the *P. expansum* isolates, are characterized. To exemplify the utility of the multi-omics approach, we compiled the gene, expression, and metabolite production data for patulin and penicillin G from all isolates (Figure 4 and Figure 5).

The patulin biosynthetic cluster was identified in R19 and Pe21 with an 86% homology match, whereas only 40% of the cluster was found in 404 and 413 using AntiSMASH. Micro-synteny analysis confirmed that R19 and Pe21 have the entire 15-gene cluster in one location within each genome. However, 404 and 413 only have a partial cluster in one location, containing the six gene orthologues for *patH*, *patG*, *patL*, *patI*, *patJ*, and *patK* (Figure 4A). The remaining orthologues with partial similarities were identified and were scattered across the other *P. chrysogenum* chromosomes (Figure 4A). There was no orthologue identified for *patF* in 404 or 413. All patulin cluster genes were expressed in R19 and Pe21 at higher levels than for any of the existing 404 and 413 orthologues (Figure 4B). The 404 *patC* orthologue did not have a corresponding transcript, and the 413 *patA* ortholog did not have corresponding transcript. The expression data for the gene clusters correlated with the patulin production in R19 and Pe21. The lack of patulin production in 404 and 413 can be definitively explained by reduced gene expression and absence of *patF* (Figure 4C). Targeted mass spectrometry confirmed patulin production for R19 and Pe21; patulin was not found at levels significantly above baseline in 404 and 413 growth media (Figure S3). Indeed, patulin peak areas were observed for each individual sample, and it can be confirmed the mean peak area shown for 404 and 413 have poor resolution and are likely isomers, adjacent peak tails, or background.

The penicillin G three-gene cluster was present in both *P. chrysogenum* isolates but absent in R19 and Pe21 (Table 4). Further analysis of the micro-synteny revealed that 404 has a possible tandem duplication for the *pcbC* gene (Figure 5A). All of the genes were expressed in 413, while 404 lacked expression of the duplicated *pcbC* region (Figure 5B). Finally, both 404 and 413 produced penicillin G (Figure 5C). Penicillin G could not be significantly resolved above baseline in R19 and Pe21 growth medium (Figure 5C), and peaks examination individually confirmed off-target readings.

## 4. Discussion

Postharvest decay and mycotoxin contamination are a major economic challenge for the pome fruit industry, especially with the rise of fungicide resistance. Recent advances have been made to improve alternative control options to mitigate postharvest disease, including the use of biocontrol products like Bio-Save^®^, formulated from *Pseudomonas syringae* [68]. The two *P. chrysogenum* isolates examined in this study, 404 and 413, were recently shown to successfully reduce blue mold decay in apple if applied prior to the aggressive *P. expansum* isolates [27]. A comparative omics-based approach was implemented to provide resources to guide future studies in fungal-fungal interactions in postharvest systems, further understand the mode of action behind the previously observed decay reduction, and to explore the production of industrially useful compounds. Genomic, transcriptomic, and metabolomic data was generated from in vitro cultures and revealed a swath of differences between the two *Penicillium* species. The results expand on differences in fungal virulence genes and SM clusters and SM production between the two *Penicillium* spp.

A recent study confirmed that *P. chrysogenum* isolates 404 and 413 cause minimal decay in apple fruit, which provides a unique opportunity to study disparities concerning infection biology using an aggressive blue mold causing species like *P. expansum* [27]. Of all the *P. expansum* virulence factors that have been confirmed to be involved in apple fruit decay via functional genetics (e.g., *creA, veA, pacC*) or enzymatic activity (e.g., PEPG1 = polygalacturonase), only four loci of the *lysM* gene family (5, 8, 11, and 16) did not have homologues in either 404 or 413. None of the missing genes have been shown to manifest in non-virulent phenotypes in *P. expansum* when deleted. Instead, the four *lysM* genes showed an increase in *P. expansum* decay when deleted, suggesting they could be negative regulators of virulence [69]. Therefore, it is unlikely that the absence of these genes is the primary contributing factor behind the observed 404 and 413 avirulence phenotype in apple fruit. Indeed, most of the *P. expansum* virulence factors had orthologues in *P. chrysogenum* which evidently do not equate to conserved functionality in *P. chrysogenum*. As expected, the in vitro data did not show stark differences in expression of the known *P. expansum* virulence genes, as growth in axenic culture does not mimic the specific apple host environment nor does it mimic apple cell physiology. However, due to the lack of growth exhibited by *P. chrysogenum* in apple, axenic culture serves as the most appropriate milieu to study differences in small molecules produced by each species that may mediate fungal-fungal interactions and/or facilitate apple fruit decay. Taken together, we hypothesize that the simple model of presence or absence of fungal virulence genes is likely not a major underpinning of *P. expansum*’s aggressive nature. Instead, the ability of *P. expansum* to aggressively decay fruit is likely due to complex gene regulation and peptide or small molecule production during apple infection, which is the subject for future investigation.

The genome sequences generated in this study are improved resources for the fungal genetics community due to their contiguous, near-chromosomal level assemblies, high read quality and sequencing depth, and improved annotation accuracy/calling due to the corresponding RNA-Seq dataset. All of the -omics data generated in this study was derived from the same culture conditions (e.g., mycelial mats from same flask were used to isolate gDNA, total RNA and broths used for metabolomics analysis), allowing accurate, streamlined comparison between data types and across fungal isolates. These genomic resources provide a strong foundation for understanding biology of two *Penicillium* spp. with contrasting fungal lifestyles. For example, it was previously found that isolates 404 and 413 had a missing gene homologue from the patulin cluster, *patF*, while R19 and Pe21 had complete patulin biosynthesis clusters [27]. However, the short-read genomes did not allow the level of resolution required for determining synteny when assembled de novo. Further, patulin production was not experimentally determined for the *P. chrysogenum* 404 and 413 isolates in that study. Here, genome mining via SM cluster analysis confirmed partial, localized patulin biosynthetic gene clusters in 404 and 413 on chromosome 2, while R19 and Pe21 had high homology of the entire, intact 15-gene cluster on *P. expansum* chromosome 4. Visualization of the partial cluster synteny in 404 and 413 supported the initial SM cluster match, as only six genes (*patGHIJKL*) were identified in a localized area. Expression of the cluster orthologues exhibited relatively higher abundance for the two *P. expansum* isolates for most of the genes. Subsequent metabolomics analyses demonstrated that both 404 and 413 do not produce patulin, whereas both *P. expansum* isolates produced copious amounts of the toxin in culture. Taken together, the integration of omics-based data for the patulin biosynthetic gene cluster provides definitive support that *P. chrysogenum* does not produce patulin, while *P. expansum* does, corroborating previous findings in the literature [27,34]. In addition, we show here for the first time the chromosomal location(s) of the patulin gene cluster in both *Penicillium* species and hypothesize that *P. chrysogenum* lost the central portion of the cluster to a recombination event during evolutionary divergence of the species that resulted in retention of only the flanking cluster genes, *patGHIJKL*.

Published *P. chrysogenum* genomic studies have shown bioinformatically and experimentally (e.g., pulse-field gel electrophoresis) that the genome is comprised of four chromosomes, consistent with the 404 and 413 assemblies in this study [66]. Further, previous comparative genomics of *P. chrysogenum* isolates showed a larger number of genetic duplications and overall larger chromosomes than other *Penicillium* species, with 13% of the gene families in *P. chrysogenum* found to be species-specific [34]. Similarly, 404 and 413 had larger chromosomes, almost 1000 more identified protein coding sequences, longer repeats (>1 Mb), and unique SM clusters identified than the R19 and Pe21 assemblies. However, many discrepancies have been observed across published representatives of *P. chrysogenum*, likely attributed, in part, to intentional selection pressure and mutagenic approaches to generate strains with high production of penicillin [66]. Here, the high similarity between the 404 and 413 isolates in genomic composition, SM cluster identification, expression, and small molecule production could be attributed to their identical geographical origin. The new R19 and Pe21 genome-level sequence assemblies also match the contig number of a recently available *P. expansum* isolate, IBT 35385, with 10 genomic contigs; however to our knowledge, no experimental confirmation of chromosome number has been demonstrated for this species (NCBI GenBank: GCA_028827855.1). Nonetheless, there were clear differences between the R19 and Pe21 expression profiles and metabolites produced in culture. Considering R19 was isolated from Pennsylvania, USA in 2011 and Pe21 from Israel in 2012 (NCBI BioSample: SAMN02928572), likely from apples with differing physiology (i.e., cultivars, maturity, storage regimens), the intraspecies differences may indicate evolutionary divergence. Indeed, as both are successful apple pathogens, the dissimilarities may further highlight niche pressures or biological variation amongst strict necrotrophs but may not represent necessities for virulence in apple fruit.

An untargeted metabolomics approach was used to putatively identify compounds produced by these two *Penicillium* species in culture. It should be noted misidentifications (e.g., isomers) could be indicated despite careful measures taken to ensure compound accuracy, as many of these molecules were not verified with purified chemical standards, nor examined for biological activity within each fungal species. Even so, many metabolites found suggest a unique fungal chemical repertoire for each of the species. For instance, multiple phytohormone-like compounds were detected exclusively in the *P. expansum* broth cultures. The capacity of the fungus to produce phytohormones, including an isomer of indole 3-acetic acid (IAA), phloroglucinol-related compounds (auxin and cytokinin-like activity), gallic acid (jasmonic acid (JA) and phenylpropanoid pathway induction), and gentisic acid (associated with salicylic acid (SA) pathway), as well as the JA precursor, 12-oxo phytodienoic acid, could suggest that the pathogen may manipulate the apple metabolism and defense response. To the best of our knowledge, this is the first indication of production of these compounds by *P. expansum* in axenic culture. Studies have shown that plant pathogens may produce phytohormones to help evade plant immune response, and to improve fungal virulence [70,71]. For example, it was confirmed that *Penicillium digitatum* can produce IAA, JA, 12-oxo phytodienoic acid, and SA, and that phytohormones are altered to facilitate infection in citrus [72]. Further, more mature apples with physiological shifts (i.e., reduced firmness, higher available sugar, etc.) have been linked to increased *P. expansum* tissue colonization [73], and *P. expansum* colonization appears to enhance ripening during infection as evidenced by increased ethylene detection [74]. Considering IAA and JA have previously been shown to stimulate ethylene production and ripening in apple fruit [75], it seems plausible that production of these compounds by *P. expansum* could aid the fungus in altering the apple fruit physiology. During *P. expansum* apple infection, ethylene-mediated defense responses are altered compared to a non-host interaction with *P. digitatum* [70]. Additionally, gallic acid levels increase in *P. expansum*-infected apples and have a concomitant increase in patulin [76]. Whether these compounds are produced to behave as or mimic phytohormones in vivo by *P. expansum*, by the apple, or both in concert during infection has yet to be determined but could significantly shift existing postharvest fungal-plant interactions paradigms to possibly leverage for new decay control mechanisms.

Among some of the most abundantly produced antimicrobials by *P. expansum* were corchorifatty acid F, perillic acid, and sorbic acid. Corchorifatty acid F is a lineolic acid derivative originally identified from *Corchorus olitorius L.* leaf tissue, and is a potential antifungal compound [77,78]. Perillic acid is a cyclohexenecarboxylic acid with antimicrobial activity against Gram-positive bacteria (*Staphylococcus aureus, Bacillus subtilis*), yeast (*Candida albicans*), and fungi (*Aspergillus brasiliensis*) that is commonly used as an antimicrobial in cosmetics [79]. Sorbic acid is a weak acid used as a food preservative with fungistatic activity that has been shown to delay germination in *Aspergillus niger* due to lowered cytosolic pH [80,81]. It is possible that *P. expansum* produces sorbic acid to compete against acid-sensitive species or utilizes sorbic acid production to stimulate the production of patulin [82]. Overall, these compounds may benefit the pathogen through competitive advantage against other sensitive organisms in the postharvest infection court. Furthermore, future biocontrol organisms used against *P. expansum* must have tolerance to these compounds, if found to be produced by the fungus during decay. Notwithstanding, utilizing *P. expansum* strains as a source of these compounds in scale up fermentation processes and/or by mining their genomes for biosynthetic clusters to move them into other organisms (e.g., yeast) for industrial scale production is of high value.

Industry has used *P. chrysogenum* for many years as a “biofactory” due to the heightened capacity of the species to produce the antibiotic penicillin [28]. Confirmation of an intact penicillin SM cluster, gene expression, and production of penicillin G was observed in 404 and 413 presented in this work. Expanding the utility of *P. chrysogenum* to produce more compounds of interest beyond penicillin could be useful to the broader fungal scientific community and industry alike [28]. The untargeted metabolomics data was mined to find additional metabolites produced by *P. chrysogenum* with natural product potential. Many metabolites with therapeutic potential were produced by *P. chrysogenum* in these conditions, including 3,8-dihydroxy-6-methyl-9-oxo-9H-xanthene-1-carboxylic acid (anticancer), 6-methoxy-2-oxo-2H-1-benzopyran-3-carboxylic acid (diuretic), archin (anti-inflammatory, antitumor), luteolin (anticancer), N-acetyldopamine (anti-inflammatory), N-acetyltyramine (antitumor), and chrysophanic acid (anticancer). Other produced antimicrobial compounds besides penicillin may contribute toward a potential role of *P. chrysogenum* as an effective biocontrol agent against a variety of microbial pathogens. For example, n-acetyltyramine has been shown to inhibit acyl-homoserine lactone-based quorum sensing systems [83] and has also demonstrated mild antifungal activity [84]. Scopoletin is a coumarin that has antioxidant and antifungal properties [85]. When produced by *Penicillium janthinellum*, it has been shown to be effective against soybean nematode while simultaneously improving soybean health [86]. Finally, some *P. chrysogenum*-produced compounds may allow for apple host recognition or induction of defense that would normally be undetected or unstimulated when *P. expansum* is present alone. Daidzein 4′-sulfate (4-(7-hydroxy-4-oxo-4H-chromen-3-yl) phenyl hydrogen sulfate), a highly abundant compound produced by isolates 404 and 413 is an isoflavone. Isoflavones, like daidzein, are linked to phenylpropanoid metabolism and defensive phytoalexins in legume plants against fungal phytopathogens, though little is known of their role in apple fruit [87,88]. This explanation does support the observed efficacy of *P. chrysogenum* correlated with advanced establishment in the apple fruit [27], however, more functional work with the compounds is warranted. The list of desirable bioactive compounds could be further expanded with changes to production conditions, such as fermentation or induction of desired biosynthesis pathways. Further, some metabolites have more than one identified biological function, further supporting production interest and need for in-depth analysis in multiple biological contexts. The prospect of metabolomic production and manipulation is now further substantiated by the plethora of omics-based resources and functional genetic tools to optimize these strains in the laboratory and at an industrial level.

Degradation, inhibition, or sequestration of virulence factors could also reduce rot and postharvest decay incited by *P. expansum*. For instance, the antifungal activity that can be overcome by some fungi (e.g., *Penicillium* spp.), and degradation of this known virulence factor was shown in vitro with *Monolinia fructicola* [16,27,89]. As isolates 404 and 413 can tolerate patulin and have partial genetic similarity to the patulin biosynthetic cluster, it is plausible they too have an existing mechanism to reduce or disable patulin [27]. Hence, *P. chrysogenum* can be explored and manipulated for use as a model system to understand genus-level patulin tolerance mechanisms in the fungus. Further, the lack of patulin production shown here in conjunction with toxin tolerance and absence of decay symptoms highlight this species as a prime candidate for biocontrol agent development against patulin-producing pathogens like *P. expansum*.

## 5. Conclusions

Blue mold is a major problem for the postharvest pome fruit storage, packing and processing industries. Current mitigation strategies are not sufficient in eliminating the disease, nor to abate the production and accumulation of the mycotoxin patulin in processed pome fruit products. Understanding the omics-based differences between virulent and nonvirulent species of *Penicillium* is one way to determine fungal genetic requirements, signaling mechanisms, and metabolomic suites necessary for apple-fruit infection. These factors can then be the main targets to serve for the development of novel decay control strategies. Additionally, through the integrated study of metabolomics, genomics, and exploring phenotypic differences between the species, the *P. chrysogenum* strains in this study have the potential to be optimized as biocontrol antagonists against blue mold decay, as contemporary laboratory tools for studying *Penicillium* spp. virulence factors, and as “fungal biofactories” to produce compounds of industrial importance.

## Figures and Tables

**Table 1:** Whole genome sequencing and de novo assembly of *P. chrysogenum* 404 and 413, and *P. expansum* R19 and Pe21.

Genome	R19 ^1^	Pe21	404	413
**Species**	*P. expansum*	*P. expansum*	*P. chrysogenum*	*P. chrysogenum*
	*Initial Assembly Statistics*			
Sequencing Platform	PacBio	Oxford Nanopore	Oxford Nanopore	Oxford Nanopore
Length (bp)	-	32,558,303	32,571,984	32,551,633
Contig Number	-	10	6	9
N50	-	5,397,789	9,112,179	6,356,666
	*Post-processing Assembly Statistics*			
Length (bp)	32,896,497	32,468,381	32,569,297	32,630,660
Contig Number	16	5	4	4
N50	8,173,214	7,894,026	9,112,601	9,112,742
GC Content	47.28%	47.48%	48.81%	48.82%
Busco Complete	96.0%	96.5%	96.8%	96.8%
Busco Complete, single copy	95.9%	96.4%	96.4%	96.4%
Busco Complete, duplicated	0.1%	0.1%	0.4%	0.4%
Busco Fragmented	1.0%	0.7%	0.5%	0.5%
Busco Missing	3.0%	2.8%	2.7%	2.7%

^1^ Using NCBI assembly accession PRJNA497398.

**Table 2:** Genome assembly comparisons for *P. chrysogenum* 404.

Assembly Software	Flye	Flye (Duplex Polishing)	HiFiasm	Verkko
Simplex sequence used	Yes	Yes	Yes	Yes
Duplex sequence used	No	Yes	Yes	Yes
Number of contigs	6	6	99	24
Total contig length (bp)	32,621,534	32,571,904	36,183,816	33,335,027
Contigs > 500 bp	6	6	99	24
Contigs > 1 Kb	6	5	99	24
Contigs > 10 Kb	6	4	74	24
Contigs > 100 Kb	4	4	27	5
Contigs > 1 Mb	4	4	15	5
N50	9,113,167	9,112,179	1,734,376	6,357,403

**Table 3:** Genome annotation of *P. chrysogenum* 404 and 413, and *P. expansum* R19 and Pe21.

Genome	R19 ^1^	Pe21	404	413
**Species**	*P. expansum*	*P. expansum*	*P. chrysogenum*	*P. chrysogenum*
	*Transposable Elements and Repeats*			
RepeatMasker	7442	7071	7140	7163
RepeatModeler	3094	2669	2971	3237
Consensus Features	10,536	9740	10,111	10,400
Total Length (bp)	1,461,084	1,685,573	2,576,305	2,836,205
	*Genes*			
Number of gene	11,911	11,326	12,246	12,774
Average gene length (bp)	1890	1661	1743	1698
Average number of exons	3.2	3.2	3.1	3.1
Busco Complete	98.2%	98.1%	98.4%	98.2%
Busco Complete, single copy	95.4%	97.9%	96.2%	96.1%
Busco Complete, duplicated	2.8%	0.2%	2.2%	2.1%
Busco Fragmented	0.9%	0.7%	0.8%	1.0%
Busco Missing	0.9%	1.2%	0.8%	0.8%
	*Non-coding Genes*			
rRNA	63	37	51	65
tRNA	204	150	156	157

^1^ Using NCBI assembly accession PRJNA497398.

**Table 4:** Percent similarity of biosynthetic gene cluster in each *Penicillium* spp. isolate ^1^.

Type	Most Similar Known Cluster	404	413	R19	Pe21
Terpene	14-(N,N-dimethylleucyloxy)paspalinine/14-(leucyloxy)paspalinine/14-hydroxypaspalinine	30	30	n.d.	n.d.
T1PKS	4-oxomacrophorin A/macrophorin A/5′-epimacrophorin B	100	100	54	54
T1PKS, NRPS	AbT1	n.d.	n.d.	100	100
T1PKS	ACT-Toxin II	n.d.	n.d.	n.d.	100
T1PKS, NRPS	andrastin A	100	100	100	100
fungal-RiPP-like	ankaflavin/monascin/rubropunctatine/monascorubrin	n.d.	n.d.	12	8
T1PKS	azasperpyranone A/azasperpyranone B/azasperpyranone C/azasperpyranone D/azasperpyranone E/azasperpyranone F/azasperpyranone G/azasperpyranone H	12	12	12	12
NRPS-like	Choline	100	100	100	100
NRPS	chrysogine	83	83	n.d.	n.d.
NRPS	chrysoxanthone A/chrysoxanthone B/chrysoxanthone C	65 (8)	65 (8)	17	17
T1PKS, terpene	Citrinin	n.d.	n.d.	56	56
NRPS, indole	Clapurines	n.d.	n.d.	72	72
T1PKS, indole	communesin A/communesin B/communesin C/communesin D/communesin E/communesin G/communesin H	n.d.	n.d.	100	100
Terpene	Conidiogenone	100	100	n.d.	n.d.
NRPS	cyclo-(D-Phe-L-Phe-D-Val-L-Val)/cyclo-(D-Tyr-L-Phe-D-Val-L-Val)/cyclo-(D-Tyr-L-Trp-D-Val-L-Val)/cyclo-(D-Phe-L-Trp-D-Val-L-Val)/cyclo-(D-Phe-L-Phe-D-Val-L-Ile)/cyclo-(D-Phe-L-Phe-D-Ile-L-Val)/cyclo-(D-Tyr-L-Trp-D-Val-L-Ile)/cyclo-(D-Tyr-L-Trp-D-Ile-L-Val)/cyclo-(D-Tyr-L-Phe-D-Val-L-Ile)/cyclo-(D-Tyr-L-Phe-D-Ile-L-Val)	n.d.	n.d.	100	n.d.
T1PKS, NRPS	Equisetin	n.d.	n.d.	45	45
NRPS, T1PKS	flavichalasine F/flavichalasine G/aspochalasin C/aspochalasin E/aspochalasin M/TMC-169/aspergillin PZ	18	18	36 (18)	36
NRPS	Gliovirin	n.d.	n.d.	62	62
NRPS	gramillin A/gramillin B	17	17	17	17
NRPS-like, NRPS, terpene	hancockiamide E/hancockiamide B/hancockiamide C/hancockiamide D/hancockiamide A/hancockiamide F	25	25	n.d.	n.d.
T1PKS	harziphilone/t22azaphilone/isoharziphilone-1/isoharziphilone-2/compound 4/compound 1	n.d.	n.d.	40	40
T1PKS	HEx-pks1 polyketide	n.d.	n.d.	60	60
NRPS, indole	histidyltryptophanyldiketopiperazine/dehydrohistidyltryptophanyldiketopiperazine/roquefortine D/roquefortine C/glandicoline A/glandicoline B/meleagrine	100	100	57	57
NRPS	loline/N-acetylnorloline/N-formylloline	n.d.	n.d.	66	66
T1PKS, NRPS	metachelin C/metachelin A/metachelin A-CE/metachelin B/dimerumic acid 11-mannoside/dimerumic acid	50 (25)	50 (25)	n.d.	n.d.
T1PKS, terpene	Neosartorin	n.d.	n.d.	57	57
NRPS	nidulanin A	75	75	n.d.	75
T1PKS	Patulin	40	40	86	86
NRPS	Penicillin	87	93	n.d.	n.d.
NRPS	Penitremane	n.d.	n.d.	17	17
NRPS, T1PKS	Phomasetin	n.d.	n.d.	85	85
NRPS, T1PKS	phyllostictine A/phyllostictine B	n.d.	n.d.	20	20
Terpene	PR-toxin	100	100	n.d.	n.d.
T1PKS	Sorbicillin	100	100	n.d.	n.d.
terpene, T1PKS	squalestatin S1	60	60	60	60
NRPS-like	viridicatumtoxin/previridicatumtoxin/5-hydroxyanthrotainin/8-O-desmethylanthrotainin	n.d.	n.d.	27	27
T1PKS	YWA1	100	100	100	100
T1PKS	Zopfiellin	16	16	n.d.	n.d.

^1^ Percentage and identification determined using the antiSMASH program. n.d. = no data result for that cluster and isolate. Parentheses indicate a second match to the same cluster was detected.

**Table 5:** Selected abundant matched compounds in *P. chrysogenum* 404 and 413 versus *P. expansum* R19 and Pe21 culture broth (compared to control PDB) ^1^.

Compound	Formula	Chemical Class	Mono-Isotopic Mass	Retention Time (min)	Library Match Score	Reference Ion	Peak Area 404/PDB	Peak Area 413/PDB	Peak Area Pe21/PDB	Peak Area R19/PDB
3,8-Dihydroxy-6-methyl-9-oxo-9H-xanthene-1-carboxylic acid	C15 H10 O6	Xanthone	286.05	7.684	80	−	**931.83**	1055.25	0.96	2.75
3-Phenyllactic acid	C9 H10 O3	Phenylpropanoic acid	166.06	6.515	99.3 ^†^	−	**6.58**	7.02	0.34	0.16
4-(7-Hydroxy-4-oxo-4H-chromen-3-yl)phenyl hydrogen sulfate	C15 H10 O7 S	Isoflavone	334.01	7.811	91.6	−	**7998.47**	**6071.54**	19.79	4.76
5-Methylcytidine	C10 H15 N3 O5	Nucleoside	257.10	3.094	91.6	+	**15.36**	10.94	0.79	0.74
5-methyl-L-uridine	C10 H14 N2 O6	Nucleoside	258.09	4.331	87.9	+	**20.08**	**14.43**	1.09	1.77
6-methoxy-2-oxo-2H-1-benzopyran-3-carboxylic acid	C11 H8 O5	Coumarin	220.04	7.762	72.1	−	**275.72**	**358.32**	2.14	9.94
Archin	C15 H10 O5	Flavenoid	270.05	9.737	96.1	−	**54.73**	**29.23**	0.86	0.78
Chrysophanic acid	C15 H10 O4	Anthraquinone	254.06	10.582	70.3	−	**40.34**	12.37	0.61	0.81
Citreorosein	C15 H10 O6	Hydroxyanthraquinone	286.05	7.172	84.5	−	**412.60**	**359.93**	27.79	3.36
Luteolin	C15 H10 O6	Flavone	286.05	8.044	88.3 ^†^	−	**222.00**	**209.18**	1.48	3.98
N-Acetyldopamine	C10 H13 N O3	Catechol	195.09	5.194	98.8 ^†^	+	**16.89**	**18.05**	1.64	1.06
N-Acetyltyramine	C10 H13 N O2	Tyramine	179.09	6.122	87.7	−	**1337.65**	**1373.87**	2.08	4.48
Penicillin G	C16 H18 N2 O4 S	Beta-lactam antibiotic	334.10	7.735	94.8 ^†^	+	**366.49**	134.66	1.15	2.49
Rubiadin	C15 H10 O4	Anthraquinone	254.06	7.811	76.1 ^†^	−	**273.48**	**198.02**	1.44	3.37
Scopoletin	C10 H8 O4	Coumarin	192.04	7.918	79	+	72.25	67.67	1.53	1.71

^1^ Bold indicates adj. *p* < 0.05. “+” = [M + H] + 1; “−” = [M − H] − 1. Match to NIST library or to mzCloud library (“^†^” demarcation).

**Table 6:** Selected abundant matched compounds in *P. expansum* Pe21 and R19 versus *P. chrysogenum* 404 and 413 culture broth (compared to control PDB) ^1^.

Compound	Formula	Chemical Class	Mono-Isotopic Mass	Retention Time (min)	Library Match Score	Reference Ion	Peak Area 404/PDB	Peak Area 413/PDB	Peak Area Pe21/PDB	Peak Area R19/PDB
12-Oxo phytodienoic acid	C18 H28 O3	Oxylipin	292.20	7.153	86.2 ^†^	+	4.11	3.51	32.80	**349.47**
3,5-dimethylorsellinic acid	C10 H12 O4	Dihydroxybenzoic acid	196.07	7.542	92.1	+	0.85	1.00	22.00	**324.74**
3-carboxy-4-methyl-5-propyl-2-furanpropionic acid	C12 H16 O5	Fuanoid fatty acid	240.10	6.72	79.2	−	0.27	1.58	1.80	**842.44**
4-Methoxycinnamic acid	C10 H10 O3	Cinnamic acid	178.06	7.54	91.8 ^†^	+	0.98	0.71	5.06	**73.42**
Citraconic acid	C5 H6 O4	Dicarboxylic acid	130.03	4.296	99.6 ^†^	−	1.84	1.48	11.64	**17.88**
Corchorifatty acid F	C18 H32 O5	Oxylipin	328.23	7.171	72.7	−	3.09	2.81	19.39	**134.01**
Gallic acid	C7 H6 O5	Trihydroxybenzoic acid	170.02	4.972	86.7	+	1.40	1.18	**513.84**	**1189.03**
Gentisic acid	C7 H6 O4	Hydroxybenzoic acid	154.03	5.534	99.6 ^†^	−	1.25	3.21	**406.10**	**716.38**
Indole-3-acetic acid	C10 H9 N O2	Carboxylic acid	175.06	5.89	84.1 ^†^	+	2.67	1.60	**14.95**	**28.70**
Patulin	C7 H6 O4	Polyketide	154.03	4.907	85	−	5.16	2.74	209.10	**259.56**
Perillic acid	C10 H14 O2	Cyclohexenecarboxylic acid	166.10	5.227	73 ^†^	−	1.54	1.42	11.16	**244.80**
Phenol	C6 H6 O	Aromatic	94.04	5.921	99.9 ^†^	−	1.24	1.29	**41.53**	**39.81**
Sorbic acid	C6 H8 O2	Hexadianoic acid	112.05	4.175	76	+	0.27	0.59	**7.58**	**9.45**

^1^ Bold indicates adj. *p* < 0.05. “+” = [M + H] + 1; “−” = [M − H] − 1. Match to NIST library or to mzCloud library (“^†^” demarcation).

## Data Availability

The whole genome sequencing data generated in this study for *P. chrysogenum* 404, 413, and *P. expansum* Pe21 are openly available in the NCBI Sequence Read Archive (SRA) with BioProject Accession PRJNA1170244. The *P. expansum* R19 whole genome sequencing data used in this study was from the existing NCBI GenBank accession GCA_004302965.1. Complete assemblies and annotations for all four genomes presented in this study are available in a Zenodo Repository DOI 10.5281/zenodo.13904012. The metabolomics mass spectrometry data files can be retrieved from massive.ucsd.edu (MSV000095703).

## Supplementary Materials

The following supporting information can be downloaded at: https://www.mdpi.com/article/10.3390/jof11010014/s1, Figure S1: Mean mycelial weight and pH of each fungal broth culture after 7 days postinoculation; Figure S2: Transcriptional heat map of single-copy virulence gene orthologues in each isolate; Figure S3: Patulin production by *P. expansum* and *P. chrysogenum* isolates in vitro; Table S1: Virulence genes within *P. expansum* and *P. chrysogenum* genomes [90,91,92,93,94,95,96,97,98,99,100,101,102,103,104,105,106,107,108,109]; Table S2: Source parameters for patulin measurement using HPLC-high resolution mass spectrometry; Table S3: Genome sequencing statistics.
