# Supplementary material for: Omics-Based Comparison of Fungal Virulence Genes, Biosynthetic Gene Clusters, and Small Molecules in Penicillium expansum and Penicillium chrysogenum
Source: J Fungi (Basel). 2024 Dec 28;11(1):14. doi: 10.3390/jof11010014 (PMC11766614; doi:10.3390/jof11010014)
Supplement: Supplementary file 1 [file jof-11-00014-s001.zip › jof-3318209-supplementary.pdf]

Supplementary Materials

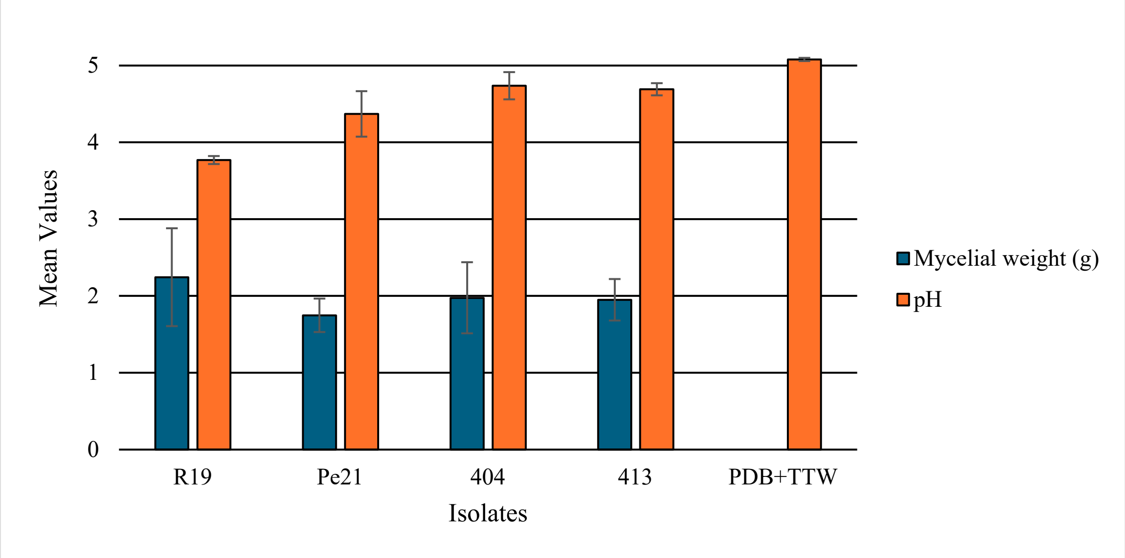

Figure S1. Mean mycelial weight (blue) and pH (orange) of each fungal broth culture after 7 days postinoculation. Means calculated from 3 replicate flasks of *P. expansum* R19, Pe21, *P. chrysogenum* 404, 413, and control broth. Error bars represent standard deviation across replicates. PDB = potato dextrose broth; TTW = Tween 20 treated water (carrier).

		Raw				Log2			
**MD8 query**	**Orthogroup_assignment**	**Pe21**	**R19**	**404**	**413**	**Pe21**	**R19**	**404**	**413**
AP1	OG0002049	8,109.5	28,193.3	24,434.3	20,990.3	12.985	14.783	14.577	14.357
Arfa	OG0007305	1,808.5	4,778.0	2,375.5	2,488.5	10.821	12.222	11.214	11.281
Blistering1	OG0005713	1,244.3	2,885.8	3,550.5	4,257.5	10.281	11.495	11.794	12.056
Bre2p	OG0004092	772.5	1,599.0	1,005.5	1,691.3	9.593	10.643	9.974	10.724
BrlA	OG0004112	6,133.5	1,179.5	2,523.3	3,032.5	12.582	10.204	11.301	11.566
CreA	OG0001902	20,552.3	25,198.0	29,081.5	39,021.3	14.327	14.621	14.828	15.252
Crt	OG0007361	14,091.3	58,067.0	40,303.3	55,615.3	13.783	15.825	15.299	15.763
GloI	OG0004661	21,766.5	17,714.3	18,737.5	17,250.5	14.410	14.113	14.194	14.074
Gst	OG0003328	28,015.0	219,971.0	121,375.3	81,165.5	14.774	17.747	16.889	16.309
kdmB	OG0001359	13,751.0	22,439.0	18,552.5	22,564.3	13.747	14.454	14.179	14.462
LaeA	OG0005075	17,537.5	55,975.5	86,853.0	103,820.8	14.098	15.773	16.406	16.664
MetR	OG0007013	455.3	22,055.0	1,908.0	2,337.8	8.831	14.429	10.898	11.191
NLP1/NIP	OG0003237	1,784.5	20,385.5	303.5	650.3	10.801	14.315	8.246	9.345
PacC	OG0003477	9,606.3	18,778.5	7,763.3	5,840.8	13.230	14.197	12.922	12.512
PeLysM10	OG0007711	1.0	1.8	1,265.8	1,511.0	0.000	0.807	10.306	10.561
PeLysM14	OG0005965	0.0	1.0	0.0	0.0		0.000		
PeLysM9	OG0003824	69.8	179.5	315.8	308.0	6.124	7.488	8.303	8.267
PePrt	OG0001025	62,484.0	135,738.8	123,828.8	96,210.0	15.931	17.050	16.918	16.554
PesA	OG0004565	1,710.3	8,249.0	22,408.0	9,814.5	10.740	13.010	14.452	13.261
PesC	OG0004649	10,382.0	25,811.3	38,566.8	35,279.8	13.342	14.656	15.235	15.107
PesF	OG0002892	7,458.0	33,670.5	10,747.8	15,573.8	12.865	15.039	13.392	13.927
RasA	OG0006393	1,655.5	6,226.5	21,452.3	31,042.3	10.693	12.604	14.389	14.922
RasB	OG0002085	3,160.0	9,232.3	3,261.5	4,381.5	11.626	13.172	11.671	12.097
RmtC	OG0006617	2,142.5	5,130.3	2,348.8	2,598.8	11.065	12.325	11.198	11.344
Sat	OG0004649	10,382.0	25,811.3	38,566.8	35,279.8	13.342	14.656	15.235	15.107
Scr2	OG0001292	7,029.8	13,658.0	253,670.8	170,974.0	12.779	13.737	17.953	17.383
Scr2	OG0006787	28,516.3	5,904.0	7,543.0	12,898.3	14.799	12.527	12.881	13.655
Sdc1	OG0001616	847.3	2,405.5	1,168.5	5,336.8	9.727	11.232	10.190	12.382
SntB	OG0002065	7,087.0	12,468.0	24,801.3	17,414.5	12.791	13.606	14.598	14.088
Spp1	OG0003487	1,101.5	1,904.8	1,846.0	2,482.5	10.105	10.895	10.850	11.278
Ste12	OG0001644	6,156.3	18,379.3	7,978.0	17,080.5	12.588	14.166	12.962	14.060
Swd1	OG0004035	1,156.0	3,218.3	1,550.0	2,267.5	10.175	11.652	10.598	11.147
Swd2	OG0006441	220.8	2,931.5	556.3	846.8	7.786	11.517	9.120	9.726
Swd3	OG0007664	704.8	1,395.8	1,216.5	1,658.3	9.461	10.447	10.249	10.695
Trx2	OG0006376	15,576.0	159,493.0	47,949.5	29,596.5	13.927	17.283	15.549	14.853
VeA	OG0005399	2,596.3	6,790.0	7,548.5	11,281.5	11.342	12.729	12.882	13.462
VelB	OG0007105	985.5	4,182.8	2,298.5	2,420.0	9.945	12.030	11.166	11.241
Wsc	OG0001300	4,611.8	25,649.3	46,863.5	58,363.8	12.171	14.647	15.516	15.833
Ypt7	OG0006882	8,295.8	24,760.5	17,550.5	21,848.8	13.018	14.596	14.099	14.415

Figure S2. Transcriptional heat map of single-copy virulence gene orthologues in each isolate. Log2 fold transcript across 3 replicate samples of Pe21, R19, 404, and 413 grown in PDB medium. Colors range from blue (low values) to red (high values). White indicates no reads detected.

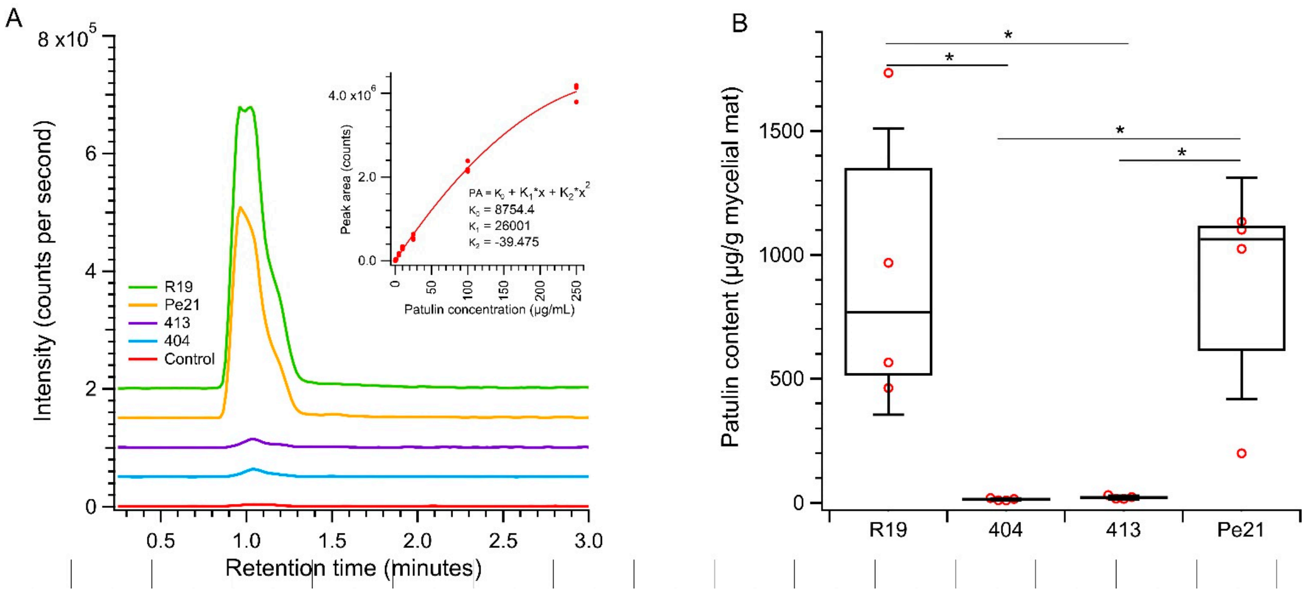

Figure S3. Patulin production by *P. expansum* and *P. chrysogenum* isolates in vitro. HPLC-high resolution mass spectrometry of *P. chrysogenum* 404, 413, *P. expansum* R19, and Pe21 broth cultures after 7 days of growth. Each sample compared to standard of patulin to determine production. A) Peak intensity of each sample compared to patulin standard. Each peak area was calculated to determine the concentration of patulin produced. B) Patulin content of each sample adjusted with the initial mycelial mat weight within each flask. Asterisk indicates significant differences based upon Tukey HSD, $p < 0.05$.

Table S1. Virulence genes within *P. expansum* and *P. chrysogenum* genomes

Gene Name	Annotation	PEX2 identifier	Virulence phenotype in vivo[1]	Ortho group assign ment	Copy Number (Pe21:R19:404:413)	Ref
AP1	Transcription factor PeAP1	PEX2_065210	Greatly reduce and significant	OG000 2049	1:1:1:1	[90]
arfA	Small GTPase superfamily, ARF/SAR type	PEX2_018890	Slight reduction and significant	OG000 7305	1:1:1:1	[91]
blistering1	Heat shock protein DnaJ, N-terminal	PEX2_008940	Slight reduction and significant	OG000 5713	1:1:1:1	[21]
bre2p	Concanavalin A-like lectin/glucanases superfamily	PEX2_051690	Moderate and significant	OG000 4092	1:1:1:1	[92]
brlA	Zinc finger, C2H2	PEX2_076900	Increase	OG000 4112	1:1:1:1	[93]
clg	Concanavalin A-like lectin/glucanase, subgroup	PEX2_031290	Moderate and significant	OG000 0297	2:2:2:2	[94]
creA	Zinc finger, C2H2	PEX2_022240	Nearly avirulent	OG000 1902	1:1:1:1	[25]
crt	Concanavalin A-like lectin/glucanase, subgroup	PEX2_105940	Slight reduction and significant	OG000 7361	1:1:1:1	[95]
eglB	Glycoside hydrolase, superfamily	PEX2_056710	Moderate and significant	OG000 0034	4:4:3:3	[96]
gloI	hypothetical protein	PEX2_019560	Slight reduction and significant	OG000 4661	1:1:1:1	[97]
god1/gox2	Glucose-methanol-choline oxidoreductase	PEX2_038230	Slight reduction and significant	OG000 0287	2:2:2:2	[95]

gst	Glutathione S-transferase/chloride channel, C-terminal	PEX2_077100	Slight reduction and significant	OG0003328	1:1:1:1	[90]
kdmB	Zinc finger, PHD-type	PEX2_000870	Moderate and significant	OG0001359	1:1:1:1	[98]
laeA	Zinc finger, C2H2	PEX2_020880	Nearly avirulent	OG0005075	1:1:1:1	[24]
lysM12	Peptidoglycan-binding Lysin subgroup	PEX2_025780	Slight reduction and significant	OG0000463	2:2:1:1	[69]
metR	hypothetical protein	PEX2_058480	Nearly avirulent	OG0007013	1:1:1:1	[99]
NLP1/NIP	Necrosis inducing protein	PEX2_080220	Slight reduction and significant	OG0003237	1:1:1:1	[18]
pacC	Zinc finger, C2H2	PEX2_077390	Nearly avirulent	OG0003477	1:1:1:1	[95]
peLysM10	hypothetical protein	PEX2_008300	Increase	OG0007711	1:1:1:1	[69]
peLysM11	Peptidoglycan-binding Lysin subgroup	PEX2_063960	Increase	OG0009123	1:1:0:0	[69]
peLysM14	Peptidoglycan-binding Lysin subgroup	PEX2_101660	Increase	OG0005965	1:1:1:1	[69]
peLysM15	Peptidoglycan-binding Lysin subgroup	PEX2_101830	Increase	OG0000066	3:3:5:1	[69]
peLysM16	hypothetical protein	PEX2_080870	Increase	OG0009152	1:1:0:0	[69]
peLysM18	Glycoside hydrolase, superfamily	PEX2_091460	Increase	OG0000463	2:2:1:1	[69]

peLysM19	Pectin lyase fold/virulence factor	PEX2_003000	Increase	OG000 0066	3:3:5:1	[69]
peLysM5	hypothetical protein	PEX2_086500	Increase	OG001 0440	1:1:0:0	[69]
peLysM7	Peptidoglycan-binding Lysin subgroup	PEX2_040510	Increase	OG000 8641	1:1:0:1	[69]
peLysM8	Peptidoglycan-binding Lysin subgroup	PEX2_060980	Increase	OG000 9994	1:1:0:0	[69]
peLysM9	Peptidoglycan-binding Lysin subgroup	PEX2_095570	Increase	OG000 3824	1:1:1:1	[69]
pepG1[2]	Glycoside hydrolase, family 28	PEX2_031340	n.d.	OG000 0228	3:3:1:1	GenBank: AF047713.1
pePrt	Peptidase S8/S53, subtilisin/kexin/sedolisin	PEX2_027670	Slight reduction and significant	OG000 1025	1:1:1:1	[100]
pesA	Phosphoadenosine phosphosulfate reductase CysH	PEX2_050620	Mild but significant	OG000 4565	1:1:1:1	[99]
pesB	sulfate anion transporter	PEX2_110150	Mild but significant	OG000 0024	3:3:4:7	[99]
pesC	Adenylylsulfate kinase	PEX2_019790	Mild but significant	OG000 4649	1:1:1:1	[99]
pesF	Transketolase, C-terminal/Pyruvate-ferredoxin oxidoreductase, domain II	PEX2_032960	Moderate and significant	OG000 2892	1:1:1:1	[99]
prx2	Redoxin	PEX2_091510	Slight reduction and significant	OG000 0334	2:2:2:2	[90]
racA	Small GTPase superfamily, Rab type	PEX2_019970	Moderate and significant	OG000 0292	2:2:2:2	[101]

rasA	Small GTPase superfamily, Rab type	PEX2_088250	Greatly reduce and significant	OG0006393	1:1:1:1	[102]
rasB	Small GTPase superfamily, Rho type	PEX2_045640	Nearly avirulent	OG0002085	1:1:1:1	[102]
rmtC	Protein arginine N-methyltransferase PRMT5	PEX2_087460	Slight reduction and significant	OG0006617	1:1:1:1	[103]
sat	Adenylylsulfate kinase	PEX2_019790	Slight reduction and significant	OG0004649	1:1:1:1	[95]
scp	Peptidase S10, serine carboxypeptidase	PEX2_089620	Moderate and significant	OG0000259	2:2:2:2	[104]
scr2	hypothetical protein	PEX2_002470	Slight reduction and significant	OG0001292	1:1:1:1	[105]
sdc1	hypothetical protein	PEX2_081070	Moderate and significant	OG0001616	1:1:1:1	[92]
sntB	Zinc finger, PHD-type	PEX2_110610	Moderate and significant	OG0002065	1:1:1:1	[23]
spp1	Zinc finger, PHD-type	PEX2_077480	Moderate and significant	OG0003487	1:1:1:1	[92]
ste12	Zinc finger, C2H2	PEX2_044670	Moderate and significant	OG0001644	1:1:1:1	[106]
swd1	hypothetical protein	PEX2_005640	Severe reduction and sig	OG0004035	1:1:1:1	[92]
swd2	hypothetical protein	PEX2_086670	Moderate and significant	OG0006441	1:1:1:1	[92]
swd3	hypothetical protein	PEX2_082610	Severe reduction and sig	OG0007664	1:1:1:1	[92]

trx2	Thioredoxin	PEX2_088120	Slight reduction and significant	OG0006376	1:1:1:1	[90]
veA	Velvet factor	PEX2_043190	Moderate and significant	OG0005399	1:1:1:1	[22]
velB	Velvet factor	PEX2_018000	Slight reduction and significant	OG0007105	1:1:1:1	[107]
wsc	Carbohydrate-binding WSC, subgroup	PEX2_002570	Slight reduction and significant	OG0001300	1:1:1:1	[108]
ypt7	Small GTPase superfamily, Rab type	PEX2_018220	Moderate and significant	OG0006882	1:1:1:1	[109]

[1]Impact on virulence listed as phenotype in vivo (apple or pear) of *P. expansum* deletion mutant strain compared to wild-type; [2]Virulence impact determined with enzyme product, not genetic mutation.

Table S2. Source parameters for patulin measurement using HPLC-high resolution mass spectrometry.

Parameter	Setting
Gas temp	350 °C
Drying gas	8 L/min
Nebulizer	35 psi
Sheath gas temp	350 °C
Sheath gas flow	11 L/min
Fragmenter	175 V
V_{cap}	5500 V
Nozzle voltage	2000 V

Table S3. Genome sequencing statistics.

Sample	Dataset	Bases (Gb)	Genome Coverage	Read N_{50} (bp)	Median Read Qscore
404	SUP simplex	10.5		8,615	18.4
404	Filtered Simplex	2.6	81	32,627	
404	Duplex	1.1		8,274	25.3
413	SUP simplex	12.6		7,925	16.5
413	Filtered Simplex	2.5	77	22,049	
413	Duplex	0.89		8,004	25.8
Pe21	HAC simplex	6.7		10,066	15.8
Pe21	Filtered Simplex	1.5	48	29,800	
Pe21	Duplex	0.34		8,246	23.4
